# Life satisfaction and happiness associated with depressive symptoms among university students: a cross-sectional study in Korea

**DOI:** 10.1186/s12991-018-0223-1

**Published:** 2018-12-18

**Authors:** Eun Hyun Seo, Seung-Gon Kim, Sang Hoon Kim, Jung Ho Kim, Jung Hyun Park, Hyung-Jun Yoon

**Affiliations:** 1 Premedical Science, College of Medicine, Chosun University, Gwangju, Republic of Korea; 2 Department of Psychiatry, Chosun University Hospital, Chosun University, Gwangju, Republic of Korea; 3 Department of Psychiatry, College of Medicine, Chosun University, 309 Pilmun-daero, Dong-gu, Gwangju, 61452 Republic of Korea

**Keywords:** Alcohol consumption, Depressive symptoms, Happiness, Life satisfaction, Sleep quality, University students

## Abstract

**Background:**

This cross-sectional study investigated the impact of life satisfaction and happiness, as well as the prevalence and correlates of depressive symptoms in a large sample of university students.

**Methods:**

We included 2338 students at 6 universities in 1 metropolitan city and 2 provinces of Korea. Depressive symptoms were assessed by the Beck Depression Inventory, and scores of 16 or higher were categorized as the presence of depression. Various sociodemographic, life satisfaction, happiness, and clinical factors (alcohol consumption and sleep quality) were measured. According to the presence of depression, sociodemographic, life satisfaction, happiness, and clinical characteristics were compared using statistical analyses. Further, a logistic regression model was constructed to examine the impact of life satisfaction, happiness, and clinical factors on depression.

**Results:**

Among participants, 13.4% were identified as having depression. Life satisfaction and happiness were associated with a lower risk of depression, while hazardous alcohol drinking and poor sleep quality were related to a higher risk of depression. In addition, female gender, subjective body shape as obese, and insufficient pocket money were found to be significant correlates of depressive symptoms.

**Conclusions:**

This study demonstrated possible risk and protective factors of underlying depressive symptoms. Especially, our findings suggest that improvement in life satisfaction and happiness would be important in the prevention and management of depression. Our findings may contribute to developing specialized mental health programs for prevention, screening, and treatment of depression among university students.

## Background

Depression is considered an important public health problem owing to its high prevalence rate [1]. It has been predicted that depression will be the second leading cause of disability by 2020 [2]. University students belong to a particular population undergoing a crucial period of transition during which they are required to make major life decisions and prepare for their future. In this critical stage of development, they may encounter diverse problems such as adjustment to a new environment away from home, financial stress, and academic difficulties. As a reaction to this stress, they may suffer from depression without realizing they are depressed. Previous studies have shown that depressive symptoms among university students are substantial around the world [3–6], and the prevalence rate appears to be increasing [7, 8]. Depression has been emphasized in university students, because depressive symptoms have a negative effect on academic performance [9] and in severe cases may even lead to suicide [10].

Previous studies on the mental health of university students have mainly investigated the prevalence of depressive or anxiety symptoms and related sociodemographic variables such as age, gender, family history of psychiatric disorders, and living or financial situation [3, 11]. Low positive emotions as well as high negative emotions are vulnerable factors for both the development and maintenance of depression [12]. The benefits of positive emotions are particularly relevant to those suffering from depression, since positive emotions have been shown to facilitate recovery from the cardiovascular effects of negative emotions [13, 14] and to buffer against relapses [15]. Nevertheless, most previous studies on depression have mainly focused on negative emotions. Contrary to traditional psychology, positive psychology is primarily concerned with positive aspects of life such as life satisfaction, happiness, gratitude, and resilience rather than psychological deficits [16]. Although life satisfaction and happiness are a broad and non-specific perception, they are important contributors to subjective well-being [17] and have been widely studied owing to their interactive effects on mental health [18, 19]. Positive cognitions and emotions have been shown to be strong predictors of happiness that alleviate automatic negative thoughts that can lead to depression [20], suggesting that higher levels of happiness may have a protective effect on depression. Conversely, dissatisfaction with life has been reported to predict depression longitudinally as well as to be associated with concurrent depressive symptoms [21]. Among the various clinical factors, alcohol consumption [22, 23] and sleep disturbance [24] have been particularly associated with depression.

Despite the research evidence concerning the significant role of positive psychology in improving mental health [16, 25], there have only been a few studies focusing on the impact of life satisfaction and happiness on depressive symptoms among university students, and the sample sizes have been relatively small. Similarly, there are limited studies on the prevalence and correlates of depressive symptoms in this population in Korea. For these reasons, we investigated the impact of life satisfaction and happiness on depressive symptoms as well as the prevalence and associated factors of depression in a large sample of university students.

## Methods

### Participants and procedure

A cross-sectional study was conducted with 2451 undergraduate students at six universities in Gwangju metropolitan city and two provinces (Jeollanam-do and Jeollabuk-do) in Korea from November 2013 to April 2014. The selection of participants was done by the convenience sampling method. Written informed consent was obtained after the aims of the study had been explained to the participants. Participants were assured that their information and responses would be confidential and anonymous. The participants then completed a questionnaire regarding sociodemographic characteristics (age, gender, grade, religion, subjective socioeconomic status, subjective body shape, and subjective pocket money), depressive symptoms, alcohol consumption, sleep quality, and positive psychological factors including life satisfaction and happiness. This study conformed to the provisions of the Declaration of Helsinki, and has been approved by the Institutional Review Board at the Chosun University (Ref. No.: 2-1041055-AB-N-01-2018-50).

### Instruments

#### Assessment of depressive symptoms

Depressive symptoms were assessed by the Beck Depression Inventory (BDI) [26]. The BDI is a 21-item instrument designed to evaluate the presence and severity of depressive symptoms. Each item is rated from 0 to 3 with a higher score representing more severe depressive symptoms. The reliability and validity of the scale have been previously confirmed [27]. Cronbach’s alpha of the BDI was 0.88 in this sample. In the present study, a cutoff score of ≥ 16 indicated the presence of depression, which was suggested to minimize the false negative rate of depression in a previous study with a Korean sample [28].

#### Assessment of clinical factors

Alcohol consumption was evaluated using the Alcohol Use Disorders Identification Test (AUDIT), which is a screening instrument designed to identify hazardous or harmful alcohol drinking [29]. It consists of ten questions related to alcohol consumption, symptoms of alcohol dependence, and problems resulting from alcohol consumption. The total score ranges from 0 to 40 with a higher score indicating more hazardous alcohol drinking. The reliability and validity of the AUDIT have been previously confirmed in Korea [30]. In this study, the cutoff score indicating the presence of hazardous alcohol drinking was 12 for males and 8 for females, which was suggested by previous studies in Korea [30, 31]. Cronbach’s alpha of the AUDIT was 0.83 in this sample.

Sleep quality was assessed by the Pittsburgh Sleep Quality Index (PSQI) [32]. The PSQI is a self-administered instrument that differentiates poor sleep from good sleep by measuring seven components including subjective sleep quality, sleep latency, sleep duration, habitual sleep efficiency, sleep disturbance, use of sleep medications, and daytime dysfunction over the last month. Each of the seven component scores is weighted equally on a scale of 0 (*no difficulty*) to 3 (*severe difficulty*). The sum of the seven component scores yields the global PSQI score. A total score of 6 or greater indicates poor sleep quality; the higher the PSQI score, the poorer the sleep quality [32]. The reliability and validity of the Korean version of the PSQI have been confirmed [33]. Cronbach’s alpha of the PSQI was 0.83 in this sample.

#### Assessment of life satisfaction and happiness

The Satisfaction with Life Scale (SWLS) was used to assess life satisfaction [34]. The SWLS is a 5-item scale assessing satisfaction with one’s life, and each item is rated from 1 (*strongly disagree*) to 7 (*strongly agree*). Higher scores indicate greater satisfaction with life. The reliability and validity of the SWLS have been confirmed [34]. Cronbach’s alpha was found to be 0.89 in this sample. Happiness was assessed by the Positive Psychotherapy Inventory (PPTI) [35], which consists of 21 items measuring three domains of happiness: pleasant life, engaged life, and meaningful life. Each item is rated from 0 to 3 with higher scores indicating a higher level of overall happiness. The Korean version of the PPTI has been previously validated [36]. Cronbach’s alpha was found to be 0.92 in this sample.

### Statistical analysis

All participants were divided into two groups (depressed or non-depressed) depending on the BDI cutoff score of 16. Group comparisons of sociodemographic characteristics, hazardous alcohol consumption, and poor sleep quality, and mean AUDIT, PSQI, SWLS, and PPTI scores were analyzed using the independent *t*-test for continuous variables and Chi square test for categorical variables. Multiple logistic regression analysis using the backward-conditional method was performed to identify life satisfaction, happiness, and clinical factors associated with depression. Provided there were significantly different sociodemographic variables between the two groups, we controlled the variables in the logistic regression model.

These analyses were performed using the Statistical Package for the Social Sciences, version 23.0 (SPSS; Chicago, IL). All statistical analyses were two-tailed with a significance level set at *p* < 0.05.

## Results

### Prevalence and sociodemographic correlates of depression

A total of 2451 students participated in the survey. Excluding 113 invalid questionnaires (those with > 20% questions unanswered), 2338 students completed the self-administered questionnaire. Of these, 1071 (45.8%) were male and 1267 (54.2%) were female. The mean age of the participants was 23.7 ± 4.2 years. The prevalence rate of experiencing depression was 13.4% (*n* = 314). Female students showed a significantly higher rate of depression than male students (male: 10.2%, female: 16.2%, *p* < 0.001). The prevalence rate of depression was significantly higher in students who perceived their body shape as obese compared to those who perceived their body shape as average or slim (obese: 16.2%, average: 10.2%, slim: 14.2%, *p* = 0.001). Depressive symptoms were also significantly higher in students who perceived the level of their pocket money was insufficient compared to those who perceived their pocket money was moderate or sufficient (insufficient: 19.1%, moderate: 10.2%, sufficient: 10.6%, *p* < 0.001). However, there were no significant differences with regard to age, grade year, religion, and socioeconomic status between the depressed and non-depressed groups. The sociodemographic characteristics of the sample and comparisons according to the presence of depression are presented in Table 1.

**Table 1:** Group comparisons of sociodemographic characteristics according to the presence of depression

Socio-demographic variables	Depressive symptoms			*t*/*χ*^2^	*p*
	No (BDI < 16) *N* (%) or mean ± SD 2024 (86.6)	Yes (BDI ≥ 16) *N* (%) or mean ± SD 314 (13.4)	Total *N* (%) or mean ± SD 2338 (100.0)		
Age (years)	23.8 ± 4.2	23.3 ± 4.0	23.7 ± 4.2	1.82	0.069
Gender					
Male	962 (47.5)	109 (34.7)	1071 (45.8)	17.99	< 0.001
Female	1062 (52.5)	205 (65.3)	1267 (54.2)		
Grade					
Freshman	346 (17.1)	58 (18.5)	404 (17.3)	2.75	0.432
Sophomore	856 (42.3)	132 (42.0)	988 (42.3)		
Junior	734 (36.3)	105 (33.4)	839 (35.9)		
Senior	87 (4.3)	19 (6.1)	106 (4.5)		
Religion					
No	1034 (51.1)	168 (53.5)	1202 (51.4)	5.60	0.231
Christianity	573 (28.3)	71 (22.6)	644 (27.5)		
Catholicism	264 (13.0)	51 (16.2)	315 (13.5)		
Buddhism	139 (6.9)	22 (7.0)	161 (6.9)		
Other religions	14 (0.7)	2 (0.6)	16 (0.7)		
Subjective body shape					
Obese	704 (34.8)	136 (43.3)	840 (35.9)	13.35	0.001
Average	772 (38.2)	88 (28.0)	860 (36.8)		
Slim	545 (27.0)	90 (28.7)	635 (27.2)		
Subjective SES					
High	72 (3.6)	7 (2.2)	79 (3.4)	3.38	0.184
Middle	1882 (93.2)	290 (92.9)	2172 (92.9)		
Low	65 (3.2)	15 (4.8)	80 (3.4)		
Subjective pocket money					
Sufficient	915 (45.3)	109 (34.7)	1024 (43.8)	33.32	< 0.001
Moderate	457 (22.6)	52 (16.6)	509 (21.8)		
Insufficient	649 (32.1)	153 (48.7)	802 (34.3)		

*BDI* Beck Depression Inventory, *SES* socioeconomic status

### Characteristics of life satisfaction, happiness, and clinical factors according to the presence of depression

The Satisfaction with Life Scale scores were significantly lower in the depressed group than non-depressed group (*p* < 0.001). Similarly, the depressed group showed significantly lower scores in all three domains of the PPTI (*p* < 0.001). Meanwhile, the clinical factors exhibited the opposite results. The rate of hazardous alcohol drinking was significantly higher in the depressed group compared to the non-depressed group (depressed group: 42.0%, non-depressed group: 35.3%, *p* = 0.021). In addition, the AUDIT scores were significantly higher in the depressed group (*p* = 0.003). In terms of sleep quality, the frequency of poor sleep was significantly higher in the depressed group (depressed group: 74.5%, non-depressed group: 40.2%, *p* < 0.001), and the PSQI total scores were significantly higher in the depressed group than in the non-depressed group (*p* < 0.001). Further, it was revealed that all 7 component scores of the PSQI were significantly higher in the depressed group than in the non-depressed group (*p* < 0.001). The comparisons of life satisfaction, happiness, and clinical factors according to the presence of depression are presented in Table 2.

**Table 2:** Group comparisons of life satisfaction, happiness, and clinical factors according to the presence of depression

Variables	Depressive symptoms			*t*/*χ*^2^	*p*
	No (BDI < 16) *N* (%) or mean ± SD	Yes (BDI ≥ 16) *N* (%) or mean ± SD	Total *N* (%) or mean ± SD		
Life satisfaction					
SWLS	22.4 ± 5.7	15.5 ± 5.5	21.5 ± 6.2	20.05	< 0.001
Happiness					
PPTI	34.5 ± 9.9	22.6 ± 8.6	32.9 ± 10.5	22.36	< 0.001
Pleasant life	12.9 ± 3.7	8.5 ± 3.5	12.3 ± 4.0	19.95	< 0.001
Engaged life	11.7 ± 3.9	7.9 ± 3.8	11.2 ± 4.1	16.08	< 0.001
Meaningful life	6.9 ± 2.8	4.6 ± 2.6	6.6 ± 2.9	13.74	< 0.001
Clinical factors					
Alcohol consumption					
AUDIT	8.1 ± 6.2	9.5 ± 8.1	8.3 ± 6.5	− 2.97	0.003
Social drinking	1309 (64.7)	182 (58.0)	1491 (63.8)	5.30	0.021
Hazardous drinking	715 (35.3)	132 (42.0)	847 (36.2)		
Sleep quality					
PSQI global score	5.1 ± 2.7	7.8 ± 3.3	5.5 ± 2.9	− 13.93	< 0.001
PSQI component score					
Sleep quality	1.0 ± 0.6	1.5 ± 0.7	1.1 ± 0.7	− 11.59	< 0.001
Sleep latency	1.3 ± 1.0	1.9 ± 1.1	1.4 ± 1.1	− 8.95	< 0.001
Sleep duration	0.9 ± 0.9	1.2 ± 1.0	0.9 ± 0.9	− 4.42	< 0.001
Sleep efficiency	0.3 ± 0.6	0.5 ± 0.8	0.3 ± 0.7	− 6.27	< 0.001
Sleep disturbance	0.8 ± 0.5	1.2 ± 0.5	0.9 ± 0.5	− 11.68	< 0.001
Hypnotic medication use	0.0 ± 0.1	0.1 ± 0.5	0.0 ± 0.2	− 8.10	< 0.001
Daytime dysfunction	0.8 ± 0.8	1.5 ± 0.9	0.9 ± 0.8	− 15.08	< 0.001
Good sleep	1210 (59.8)	80 (25.5)	1290 (55.2)	129.34	< 0.001
Poor sleep	814 (40.2)	234 (74.5)	1048 (44.8)		

*BDI* Beck Depression Inventory, *AUDIT* Alcohol Use Disorders of Identification Test, *PSQI* Pittsburgh Sleep Quality Index, *SWLS* Satisfaction with Life Scale, *PPTI* Positive Psychotherapy Inventory

### Impact of life satisfaction, happiness, and clinical factors on depression

To quantify the factors associated with depression, we conducted multivariate logistic regression analysis. Because gender, subjective body shape, and subjective level of pocket money differed significantly between the two groups, we entered these variables in the first block to control for their effects. Then, SWLS, PPTI, AUDIT, and PSQI scores were entered in the second block. After controlling for the effects of gender, subjective body shape, and subjective level of pocket money, all the positive psychological and clinical variables were significant in the final model (Table 3). SWLS (OR = 0.881, 95% CI 0.837–0.891, *p* < 0.001), PPTI (OR = 0.908, 95% CI 0.891–0.925, *p* < 0.001), AUDIT (OR = 1.027, 95% CI 1.006–1.050, *p* = 0.013), and PSQI (OR = 1.259, 95% CI 1.196–1.324, *p* < 0.001) scores were each significantly associated with depression.

**Table 3:** Logistic regression analysis for factors associated with depression

Variables	Step 1^a^					Step 2^b^				
	*B*	SE	Wald	*p*	OR (95% CI)	*B*	SE	Wald	*p*	OR (95% CI)
Gender	− 0.439	0.130	11.448	0.001	0.645 (0.500–0.831)	− 0.407	0.159	6.587	0.010	0.665 (0.488–0.908)
Subjective body shape	− 0.054	0.079	0.471	0.493	0.947 (0.811–1.106)	− 0.071	0.092	0.594	0.441	0.932 (0.778–1.115)
Subjective pocket money	0.322	0.071	20.536	< 0.001	1.379 (1.200–1.585)	− 0.073	0.088	0.684	0.408	0.932 (0.782–1.105)
SWLS						− 0.146	0.016	85.861	< 0.001	0.881 (0.837–0.891)
PPTI						− 0.097	0.009	104.291	< 0.001	0.908 (0.891–0.925)
AUDIT						0.027	0.011	6.160	0.013	1.027 (1.006–1.050)
PSQI						0.230	0.026	78.822	< 0.001	1.259 (1.196–1.324)

*AUDIT* Alcohol Use Disorders of Identification Test, *PSQI* Pittsburgh Sleep Quality Index, *SWLS* Satisfaction with Life Scale, *PPTI* Positive Psychotherapy Inventory, *CI* confidence interval, *OR* odds ratio, *SE* standard error

^a^ *χ*^2^ of model = 41.161, *df *= 3, Nagelkerke *R*^2^ = 0.031

^b^ *χ*^2^ of model = 628.838, *df *= 7, Nagelkerke *R*^2^ = 0.454

## Discussion

In the current study, we investigated the impact of life satisfaction and happiness as well as the current status and correlates of depressive symptoms in a large sample of Korean university students. Our study demonstrated that life satisfaction and happiness were significantly associated with a lower risk of depression, whereas hazardous alcohol consumption and poor sleep quality were related to a higher risk of depression. Our results also showed specific sociodemographic correlates of depressive symptoms.

Our study indicated that 13.4% of the sample was experiencing depression; a finding that is similar to previous studies with Korean and Chinese university students [5, 37]. Meanwhile, the rate is lower than what has been found in some other studies including samples from the United States and European and Middle Eastern countries reporting prevalence rates of 25–29% [38, 39] and 30–33% in meta-analyses [40, 41]. Koreans have exhibited a higher diagnostic threshold for major depression than have Americans, which results in a lower prevalence of major depression [42]. Koreans are more likely to express the symptoms of fatigue and concentration difficulty, but are less likely to express depressed mood and suicidal ideation than Americans. Hence, cross-cultural differences may influence a lower prevalence rate of depression. In addition, the different scales may partly account for the differences found among studies, because commonly used scales have fewer common and specific factors of depressive symptoms than expected [43]. Regardless of such differences, our results suggest that a substantial number of students experience depression. Therefore, active efforts to evaluate underlying depression should be encouraged, to improve the mental health of students.

Students with depression showed a lower level of life satisfaction and happiness than non-depressed students. Our logistic regression analysis further indicated that individuals with higher levels of life satisfaction and happiness had a decreased probability of having depression. As Seligman pointed out, positive psychology includes various aspects such as well-being and satisfaction (past); flow, joy, the sensual pleasures, and happiness (present); and constructive cognitions about the future [44]. In the current study, we adopted major components of past and present aspects of positive psychology. Our findings suggest that past and present aspects of positive psychology can play an important role in prevention of depression. In line with the results of the current study, it was found that life satisfaction negatively correlated with depressive symptoms in students [45]. Meanwhile, in a previous study using a large cohort sample of middle-aged people, the absence of psychological well-being was a significant risk factor for subsequent depression [46].

These previous studies and our results provide evidence regarding the protective role of life satisfaction and happiness on depression among university students. A promising approach to enhance well-being, positive psychological interventions (PPI) that aim to foster positive cognitions, emotions, or behaviors have been applied to treat a variety of psychiatric disorders [47, 48]. PPI can be particularly useful for depression characterized by a paucity of positive emotions, life meanings, and engagements [47]. A meta-analytic review found that PPI improved well-being and decreased depressive symptoms [49]. Our findings suggest that PPI, focusing on the improvement of life satisfaction and happiness, can be applied to depression in clinical practice. Interestingly, participants who had a depressed mood and voluntarily participated were more likely to benefit from PPI, implying that providing motivation and encouragement may be important in the implementation of PPI. Taken together, development and application of the life satisfaction and happiness-focused novel mental health programs addressing self-motivation may contribute to the prevention and management of depression on college campuses.

Alcohol use is common among university students, often leading to alcohol use disorders such as alcohol abuse and dependence [50]. Alcohol use and depression may be closely associated because individuals who suffer from one disorder are susceptible to the other [23]. Results of our study support this bidirectional relationship. Further, logistic regression analysis indicated that higher levels of hazardous alcohol consumption increased the probability of having depression. It has been shown that interventions including motivational interviewing and personalized normative feedback are effective modalities for reducing alcohol drinking in students [51]. Considering the high prevalence of hazardous alcohol use [52] and its significant effect on depression, efforts to perform screening and intervention programs for alcohol abuse are warranted on campuses. With regard to sleep quality, we found that individuals with poorer sleep quality had an increased probability of having depression. Similarly, previous research found that sleep problems indicated an underlying depression in university students [53], and there is increasing evidence that suggests chronic insomnia is a strong risk factor for the development of depression [24]. Moreover, our results revealed that all individual components of the PSQI, as well as global sleep quality were significantly associated with depressive symptoms. This suggests that diverse aspects of sleep quality including sleep latency, sleep duration, sleep efficiency, sleep disturbance, use of sleep medication, and daytime dysfunction may influence depression in students. Because hazardous alcohol use and sleep disturbances are each associated with depressive symptoms, students who have both may be at an especially high risk for depression. For the university health service, this point should be considered as an important issue.

The prevalence rate of depression was significantly higher in female students than in male students, which is consistent with a higher lifetime prevalence of depression found in women among the general population [1]. This is in line with findings of previous studies reporting that female students had a higher rate of depressive symptoms than male students [4, 54]. As one explanation for this finding, a cross-national study of health proposed that gender differences are greater in countries with greater gender inequalities regarding political rights and job opportunities [55]. However, some researchers have found no significant difference according to gender [5, 38], indicating the reasons for the inconsistency need to be further investigated.

We found that depressive symptoms were associated with perception of body shape as being obese. Koreans are thought to be preoccupied with body image, regardless of actual body weight [56]. Moreover, social norms overemphasize maintaining thinness as a focus of self-presentation in Korea [56]. Therefore, Korean people who perceive their body shape to be obese are likely to be more dissatisfied with their body image compared to those who do not perceive themselves as such. In this regard, a previous study revealed that body image concern, especially obesity, was a mediating factor in the relationship between body weight and depressive symptoms among Korean adults [57]. However, studies focusing on the association of body image concern with depression in university students are limited. Therefore, longitudinal studies are needed to elucidate the role of body image concern on depression among students.

Previous studies have consistently reported that lower socioeconomic status was a significant risk factor for depressive symptoms in university students [5, 54]. Interestingly, in our study, the level of subjective pocket money was found to be a significant correlate of depressive symptoms, whereas subjective socioeconomic status was not. In fact, satisfaction or dissatisfaction with economic circumstances has been well established for its significant role in mental health [58]. In addition, one’s personal financial condition such as pocket money may be different from subjective socioeconomic status. Our findings imply that personal financial difficulties rather than simply subjective socioeconomic status would be a more important correlate of current depressive symptoms in students.

This study has several limitations and future research should address them. Firstly, because the design of this study was cross-sectional, it is difficult to make causal inferences. A prospective longitudinal study is needed to confirm the causal relationships suggested by our findings. Secondly, we evaluated depression using the BDI without the clinical interview. Additional clinical interviews with those scoring above a cutoff score of the BDI would be helpful to elaborate factors associated with depression in future studies. Thirdly, there is the potential of sampling bias because the selection of participants was based on convenience sampling, limiting the generalizability of our findings. Nevertheless, the large sample size and the use of a validated scale to identify depressive symptoms might have ensured the validity of the results. Fourthly, objective data regarding body image and economic status were not obtained. Moreover, this study did not evaluate specific and major issues related to university students such as school life, academic performance, anxiety, and suicide. Thus, further study on these issues is needed to develop specialized mental health program for university students. Finally, due to the nature of self-report measures, the results of this study should be interpreted cautiously. However, participants were assured confidentiality by anonymous questionnaires, which can reduce the possibility of dishonesty. Regardless of these limitations, the present study could provide valuable information regarding the current status and associated factors of depression among university students.

## Conclusions

In conclusion, this study revealed that life satisfaction and happiness were associated with a lower risk of depression in university students. Although the relationship between various positive psychological factors and depression is not yet fully understood, our findings emphasize that enhancing life satisfaction and happiness may be important in the prevention and management of depression among students. In addition, this study identified the current status of, and factors associated with depressive symptoms among university students in Korea. This study has several important implications for administrators of universities when they are considering the sociodemographic, positive psychological, and clinical factors in dealing with the mental health issues of students. Furthermore, our findings may contribute to developing mental health programs for facilitating primary prevention, early detection, and treatment of depression as well as promoting well-being among students.

## Abbreviations

AUDIT: the Alcohol Use Disorders Identification Test
BDI: the Beck Depression Inventory
PPTI: the Positive Psychotherapy Inventory
PSQI: the Pittsburgh Sleep Quality Index
SWLS: The Satisfaction with Life Scale

## References

[1] Kessler RC, Berglund P, Demler O, Jin R, Koretz D, Merikangas KR (2003). The epidemiology of major depressive disorder: results from the national comorbidity survey replication (NCS-R). JAMA.

[2] Murray CJ, Lopez AD (1997). Alternative projections of mortality and disability by cause 1990–2020: global burden of disease study. Lancet.

[3] Eisenberg D, Gollust SE, Golberstein E, Hefner JL (2007). Prevalence and correlates of depression, anxiety, and suicidality among university students. Am J Orthopsychiatry.

[4] Mikolajczyk RT, Maxwell AE, El Ansari W, Naydenova V, Stock C, Ilieva S (2008). Prevalence of depressive symptoms in university students from Germany, Denmark, Poland and Bulgaria. Soc Psychiatry Psychiatr Epidemiol.

[5] Chen L, Wang L, Qiu XH, Yang XX, Qiao ZX, Yang YJ (2013). Depression among Chinese university students: prevalence and socio-demographic correlates. PLoS ONE.

[6] Arslan G, Ayranci U, Unsal A, Arslantas D (2009). Prevalence of depression, its correlates among students, and its effect on health-related quality of life in a Turkish university. Ups J Med Sci.

[7] Buchanan JL (2012). Prevention of depression in the college student population: a review of the literature. Arch Psychiatr Nurs.

[8] Reavley N, Jorm AF (2010). Prevention and early intervention to improve mental health in higher education students: a review. Early Interv Psychiatry.

[9] Hysenbegasi A, Hass SL, Rowland CR (2005). The impact of depression on the academic productivity of university students. J Ment Health Policy Econ.

[10] Nemeroff CB, Compton MT, Berger J (2001). The depressed suicidal patient. Assessment and treatment. Ann NY Acad Sci.

[11] Bayram N, Bilgel N (2008). The prevalence and socio-demographic correlations of depression, anxiety and stress among a group of university students. Soc Psychiatry Psychiatr Epidemiol.

[12] Van Beveren ML, Harding K, Beyers W, Braet C (2018). Don’t worry, be happy: the role of positive emotionality and adaptive emotion regulation strategies for youth depressive symptoms. Br J Clin Psychol.

[13] Fredrickson BL, Levenson RW (1998). Positive emotions speed recovery from the cardiovascular sequelae of negative emotions. Cogn Emot.

[14] Tugade MM, Fredrickson BL (2004). Resilient individuals use positive emotions to bounce back from negative emotional experiences. J Pers Soc Psychol.

[15] Fava GA, Ruini C (2003). Development and characteristics of a well-being enhancing psychotherapeutic strategy: well-being therapy. J Behav Ther Exp Psychiatry.

[16] Seligman ME, Steen TA, Park N, Peterson C (2005). Positive psychology progress: empirical validation of interventions. Am Psychol.

[17] Diener E (1984). Subjective well-being. Psychol Bull.

[18] Diener E (2000). Subjective well-being. The science of happiness and a proposal for a national index. Am Psychol.

[19] Tay L, Kuykendall L (2013). Promoting happiness: the malleability of individual and societal subjective wellbeing. Int J Psychol.

[20] Lightsey OR (1994). “ Thinking positive” as a stress buffer: the role of positive automatic cognitions in depression and happiness. J Couns Psychol.

[21] Koivumaa-Honkanen H, Kaprio J, Honkanen R, Viinamäki H, Koskenvuo M (2004). Life satisfaction and depression in a 15-year follow-up of healthy adults. Soc Psychiatry Psychiatr Epidemiol.

[22] Weitzman ER (2004). Poor mental health, depression, and associations with alcohol consumption, harm, and abuse in a national sample of young adults in college. J Nerv Ment Dis.

[23] Jané-Llopis E, Matytsina I (2006). Mental health and alcohol, drugs and tobacco: a review of the comorbidity between mental disorders and the use of alcohol, tobacco and illicit drugs. Drug Alcohol Rev.

[24] Ohayon MM, Roth T (2003). Place of chronic insomnia in the course of depressive and anxiety disorders. J Psychiatr Res.

[25] Slade M (2010). Mental illness and well-being: the central importance of positive psychology and recovery approaches. BMC Health Serv Res.

[26] Beck AT, Ward CH, Mendelson M, Mock J, Erbaugh J (1961). An inventory for measuring depression. Arch Gen Psychiatry.

[27] Beck AT, Steer RA, Carbin MG (1988). Psychometric properties of the Beck Depression Inventory: twenty-five years of evaluation. Clin Psychol Rev.

[28] Shin MS, Kim ZS, Park KB (1993). The cut-off score for the Korean version of Beck Depression Inventory. Korean J Clin Psychol.

[29] Saunders JB, Aasland OG, Babor TF, del la Fuente JR, Grant M (1993). Development of the alcohol use disorders identification test (AUDIT): WHO collaborative project on early detection of persons with harmful alcohol consumption-II. Addiction.

[30] Lee BO, Lee CH, Lee PG, Choi MJ, Namkoong K (2000). Development of Korean version of alcohol use disorders identification test (AUDIT-K): its reliability and validity. J Korean Acad Addict Psychiatry.

[31] Joe KH, Chai SH, Park A, Lee HK, Shin IH, Min SH (2009). Optimum cut-off score for screening of hazardous drinking using the Korean version of alcohol use disorder identification test (AUDIT-K). J Korean Acad Addict Psychiatry.

[32] Buysse DJ, Reynolds CF, Monk TH, Berman SR, Kupfer DJ (1989). The Pittsburgh Sleep Quality Index: a new instrument for psychiatric practice and research. Psychiatry Res.

[33] Sohn SI, Kim DH, Lee MY, Cho YW (2012). The reliability and validity of the Korean version of the Pittsburgh Sleep Quality Index. Sleep Breath.

[34] Diener E, Emmons RA, Larsen RJ, Griffin S (1985). The satisfaction with life scale. J Pers Assess.

[35] Guney S (2011). The Positive Psychotherapy Inventory (PPTI): reliability and validity study in Turkish population. Proc Soc Behav Sci.

[36] Yoon S, Sin H (2010). Validation study of the Korean version of positive psychotherapy inventory. Korean J Counsel Psychother.

[37] Roh MS, Jeon HJ, Lee HW, Lee HJ, Han SK, Hahm BJ (2006). Depressive disorders among the college students: prevalence, risk factors, suicidal behaviors and dysfunctions. J Korean Neuropsychiatr Assoc.

[38] Bostanci M, Ozdel O, Oguzhanoglu NK, Ozdel L, Ergin A, Ergin N (2005). Depressive symptomatology among university students in Denizli, Turkey: prevalence and sociodemographic correlates. Croat Med J.

[39] Mahmoud JS, Staten R, Hall LA, Lennie TA (2012). The relationship among young adult college students’ depression, anxiety, stress, demographics, life satisfaction, and coping styles. Issues Ment Health Nurs.

[40] Ibrahim AK, Kelly SJ, Adams CE, Glazebrook C (2013). A systematic review of studies of depression prevalence in university students. J Psychiatr Res.

[41] Sarokhani D, Delpisheh A, Veisani Y, Sarokhani MT, Manesh RE, Sayehmiri K (2013). Prevalence of depression among university students: a systematic review and meta-analysis study. Depress Res Treat.

[42] Chang SM, Hahm BJ, Lee JY, Shin MS, Jeon HJ, Hong JP (2008). Cross-national difference in the prevalence of depression caused by the diagnostic threshold. J Affect Disord.

[43] Shafer AB (2006). Meta-analysis of the factor structures of four depression questionnaires: beck, CES-D, Hamilton, and Zung. J Clin Psychol.

[44] Seligman ME, Snyder CR, Lopez SJ (2002). Positive psychology, positive prevention, and positive therapy. Handbook of positive psychology.

[45] Samaranayake CB, Fernando AT (2011). Satisfaction with life and depression among medical students in Auckland, New Zealand. NZ Med J.

[46] Wood AM, Joseph S (2010). The absence of positive psychological (eudemonic) well-being as a risk factor for depression: a ten year cohort study. J Affect Disord.

[47] Seligman ME, Rashid T, Parks AC (2006). Positive psychotherapy. Am Psychol.

[48] Fava GA, Ruini C, Rafanelli C, Finos L, Salmaso L, Mangelli L (2005). Well-being therapy of generalized anxiety disorder. Psychother Psychosom.

[49] Sin NL, Lyubomirsky S (2009). Enhancing well-being and alleviating depressive symptoms with positive psychology interventions: a practice-friendly meta-analysis. J Clin Psychol.

[50] Hingson RW, Heeren T, Winter MR (2006). Age of alcohol-dependence onset: associations with severity of dependence and seeking treatment. Pediatrics.

[51] Carey KB, Scott-Sheldon LA, Carey MP, DeMartini KS (2007). Individual-level interventions to reduce college student drinking: a meta-analytic review. Addict Behav.

[52] Blanco C, Okuda M, Wright C, Hasin DS, Grant BF, Liu SM (2008). Mental health of college students and their non-college-attending peers: results from the National Epidemiologic Study on Alcohol and Related Conditions. Arch Gen Psychiatry.

[53] Eller T, Aluoja A, Vasar V, Veldi M (2006). Symptoms of anxiety and depression in Estonian medical students with sleep problems. Depress Anxiety.

[54] Roh MS, Jeon HJ, Kim H, Han SK, Hahm BJ (2010). The prevalence and impact of depression among medical students: a nationwide cross-sectional study in South Korea. Acad Med.

[55] Torsheim T, Ravens-Sieberer U, Hetland J, Välimaa R, Danielson M, Overpeck M (2006). Cross-national variation of gender differences in adolescent subjective health in Europe and North America. Soc Sci Med.

[56] Han M (2003). Body image dissatisfaction and eating disturbance among Korean college female students: relationships to media exposure, upward comparison, and perceived reality. Commun Stud..

[57] Brewis AA, Han SY, SturtzSreetharan CL (2017). Weight, gender, and depressive symptoms in South Korea. Am J Hum Biol.

[58] Roberts R, Golding J, Towell T, Weinreb I (1999). The effects of economic circumstances on British students’ mental and physical health. J Am Coll Health.

